# A pilot randomized controlled trial of 7 versus 14 days of antibiotic treatment for bloodstream infection on non-intensive care versus intensive care wards

**DOI:** 10.1186/s13063-019-4033-9

**Published:** 2020-01-15

**Authors:** Nick Daneman, Asgar H. Rishu, Ruxandra Pinto, Yaseen Arabi, Emilie P. Belley-Cote, Robert Cirone, Mark Downing, Deborah J. Cook, Richard Hall, Shay McGuinness, Lauralyn McIntyre, John Muscedere, Rachael Parke, Steven Reynolds, Benjamin A. Rogers, Yahya Shehabi, Phillip Shin, Richard Whitlock, Robert A. Fowler, on behalf of the Canadian Critical Care Trials Group

**Affiliations:** 1 Division of Infectious Diseases & Clinical Epidemiology, Department of Medicine Sunnybrook Health Sciences Centre, University of Toronto and Adjunct Scientist, Institute for Clinical Evaluative Sciences, Sunnybrook Health Sciences Centre, 2075 Bayview Ave, Toronto, ON M4N 3M5 Canada; 2 Department of Critical Care Medicine, Sunnybrook Health Sciences Center, Toronto, ON Canada; 3 College of Medicine, King Saud Bin Abdulaziz University for Health Sciences, King Abdullah International Medical Research Center, Intensive Care Department, Ministry of National Guard Health Affairs, Riyadh, Kingdom of Saudi Arabia; 4 Division of Cardiology, Department of Medicine, McMaster University, Hamilton, ON Canada; 5 Division of Critical Care, St. Joseph’s Health Centre, Toronto, ON Canada; 6 Division of Infectious Diseases, St. Joseph’s Health Centre, Toronto, ON Canada; 7 Division of Critical Care Medicine, Department of Medicine, McMaster University, Hamilton, ON Canada; 8 Departments of Critical Care Medicine and Anesthesiology, Pain Management and Perioperative Medicine, Dalhousie University, Halifax, NS Canada; 9 Cardiothoracic and Vascular Intensive Care Unit, Auckland City Hospital, Auckland, New Zealand; 10 Division of Critical Care, Department of Medicine, The Ottawa Hospital, Ottawa, ON Canada; 11 Department of Critical Care Medicine, Queen’s University, Kingston, ON Canada; 12 The University of Auckland, Auckland, New Zealand; 13 Department of Biophysiology and Kinesiology, Simon Fraser University, Burnaby, BC Canada; 14 Centre for Inflammatory Diseases, Monash University School of Clinical Sciences, Clayton, Victoria, Australia; Monash Infectious Diseases, Monash Health, Clayton, VIC Australia; 15 Critical Care and Perioperative Medicine, School of Clinical Sciences, Monash University and Monash Health, Clayton, Victoria, Australia and the Clinical School of Medicine, University of New South Wales, Randwick, NSW Australia; 16 Department of Medicine and Critical Care, North York General Hospital, Toronto, ON Canada; 17 Department of Surgery, McMaster University, Hamilton, ON Canada; 18 Departments of Medicine and Critical Care Medicine, Sunnybrook Health Sciences Center, Adjunct Scientist, Institute for Clinical Evaluative Sciences, Institute of Health Policy, Management and Evaluation, University of Toronto, 2075 Bayview Ave, Toronto, ON M4N 3M5 Canada

**Keywords:** Bacteremia, Bloodstream infection, Critical care, Intensive care, Duration of treatment

## Abstract

**Background:**

The optimal treatment duration for patients with bloodstream infection is understudied. The Bacteremia Antibiotic Length Actually Needed for Clinical Effectiveness (BALANCE) pilot randomized clinical trial (RCT) determined that it was feasible to enroll and randomize intensive care unit (ICU) patients with bloodstream infection to 7 versus 14 days of treatment, and served as the vanguard for the ongoing BALANCE main RCT. We performed this BALANCE-Ward pilot RCT to examine the feasibility and impact of potentially extending the BALANCE main RCT to include patients hospitalized on non-ICU wards.

**Methods:**

We conducted an open pilot RCT among a subset of six sites participating in the ongoing BALANCE RCT, randomizing patients with positive non-*Staphylococcus aureus* blood cultures on non-ICU wards to 7 versus 14 days of antibiotic treatment. The co-primary feasibility outcomes were recruitment rate and adherence to treatment duration protocol. We compared feasibility outcomes, patient/pathogen characteristics, and overall outcomes among those enrolled in this BALANCE-Ward and prior BALANCE-ICU pilot RCTs. We estimated the sample size and non-inferiority margin impacts of expanding the BALANCE main RCT to include non-ICU patients.

**Results:**

A total of 134 patients were recruited over 47 site-months (mean 2.9 patients/site-month, median 1.0, range 0.1–4.4 patients/site-month). The overall recruitment rate exceeded the BALANCE-ICU pilot RCT (mean 1.10 patients/site-month, *p* < 0.0001). Overall protocol adherence also exceeded the adherence in the BALANCE-ICU pilot RCT (125/134, 93% vs 89/115, 77%, *p* = 0.0003). BALANCE-Ward patients were older, with lower Sequential Organ Failure Assessment scores, and higher proportions of infections caused by *Escherichia coli* and genito-urinary sources of bloodstream infection. The BALANCE-Ward pilot RCT patients had an overall 90-day mortality rate of 17/133 (12.8%), which was comparable to the 90-day mortality rate in the ICU pilot RCT (17/115, 14.8%) (*p* = 0.65). Simulation models indicated there would be minimal sample size and non-inferiority margin implications of expanding enrolment to increasing proportions of non-ICU versus ICU patients.

**Conclusion:**

It is feasible to enroll non-ICU patients in a trial of 7 versus 14 days of antibiotics for bloodstream infection, and expanding the BALANCE RCT hospital-wide has the potential to improve the timeliness and generalizability of trial results.

**Trial registration:**

Clinicaltrials.gov, NCT02917551. Registered on September 28, 2016.

## Background

The World Health Organization has declared antibiotic resistance a global public health threat, based on rising rates of resistant pathogens and diminishing rates of new antibiotic development [1]. Antimicrobial stewardship is a cornerstone of efforts to counter this threat. However, evidence-informed stewardship treatment decisions for patients with life-threatening illnesses such as bloodstream infections are challenging because little evidence exists for the optimal duration of treatment. Among patients with suspected bloodstream infections, broad-spectrum antibiotics must be initiated empirically because early adequate empiric treatment is associated with improved survival [2, 3]. Due to the rising prevalence of resistant organisms, the tailoring or de-escalation of these empiric regimens is not possible even when blood culture and susceptibility results become available. Patients must then remain on broad-spectrum agents for their full treatment course [4]. Therefore, shortening total treatment durations may be the most feasible approach to minimize patient-level and societal-level antimicrobial harms [5].

Our systematic review, national practice survey, and observational studies have documented a lack of evidence to guide optimal treatment durations for bloodstream infections, wide variation in clinical practice, and collective equipoise for a trial of 7 versus 14 days of antibiotic treatment for patients with bloodstream infections [6–8]. Through the Bacteremia Antibiotic Length Actually Needed For Clinical Effectiveness (BALANCE) pilot randomized controlled trial (ClinicalTrials.gov NCT02261506) we documented the feasibility of this trial design among 115 patients in intensive care units (ICUs) [9]. These patients served as a vanguard for the BALANCE main trial (ClinicalTrials.gov NCT03005145), which has recruited more than 600 patients across a growing number of ICU sites and countries.

The Canadian Critical Care Trials Group (CCCTG) and Australian & New Zealand Intensive Care Society Clinical Trials Group (ANZICS CTG) began the BALANCE trial in the ICU setting. As the majority of patients with bacteremia are cared for on general medical and surgical wards, we began to explore hospital-wide expansion to the full population of hospitalized patients with bacteremia as a means to improve the generalizability and timeliness of the BALANCE RCT. We first conducted a distinct BALANCE pilot trial focused on patients admitted to general hospital wards at the BALANCE central study site. We then expanded this approach to several community and academic hospitals participating in the BALANCE trial (ClinicalTrials.gov NCT02917551).

The objectives of this multi-centre BALANCE-Ward pilot RCT were three-fold: (1) to test the feasibility of ward (non-ICU) recruitment into this trial; (2) to compare the patient, pathogen, and outcome characteristics among patients enrolled in the BALANCE-Ward pilot RCT to characteristics in the prior BALANCE-ICU pilot RCT; and (3) to estimate the sample size and non-inferiority margin impacts of merging the BALANCE-Ward pilot with the BALANCE main trial.

## Methods

### General study design

We conducted a pilot RCT of 7 versus 14 days of antibiotic treatment for patients with bloodstream infection, which was identical to our prior BALANCE-ICU pilot RCT [9, 10], except that it focused on patients admitted to general medical and surgical wards. In this BALANCE-Ward pilot trial, as per the prior BALANCE-ICU pilot trial focused on critically ill patients, randomization was determined through a central, web-based system (http://www.randomize.net) with variable block sizes of four to six patients, stratified by site. The intervention related only to the duration of treatment, with patients randomized 1:1 in parallel to 7 versus 14 days of treatment. All other aspects of care (antibiotic selection, doses, intervals, routes of delivery, and timing of hospital discharge) were at the discretion of the clinical team. Participant and clinician blinding and placebo controls were not used given the diversity of pathogens and underlying foci of infection, but allocation concealment was maintained until the seventh day of treatment to mitigate selection bias and differential treatment. The central study team and statistician were blinded to treatment group. The BALANCE-Ward pilot trial was registered separately on Clinicaltrials.gov (NCT02917551), with unique ethics approval at all participating sites, so that enrolled patients could be kept distinct from the main trial until completion of the pilot and evaluation of feasibility.

### Study setting

The BALANCE-Ward pilot trial was launched at Sunnybrook Health Sciences Centre (SHSC) in October 2016, and then after 1 year extended to five other active BALANCE sites, including The Ottawa Hospital (TOH), Kingston General Hospital (KGH), Hamilton General Hospital (HGH), St. Joseph’s Health Centre (SJHC) Toronto, and North York General Hospital (NYGH).

### Inclusion/exclusion criteria

The inclusion criteria differed, by definition, from the prior BALANCE pilot RCT [9, 10] in that we considered all adult patients with a blood culture reported as positive with a pathogenic bacterium while on a *non*-ICU ward rather than reported as positive while *in an ICU*. However, the exclusion criteria were unchanged from the BALANCE pilot RCT: previously enrolled patients, those with neutropenia, organ transplantation, prosthetic valves, endovascular grafts, suspected or documented syndromes requiring prolonged treatment (endocarditis, osteomyelitis, undrained abscess, unremoved prosthetic infection), patients with a single positive culture of a common contaminant organism, or bloodstream infection with *Staphylococcus aureus, Staphylococcus lugdunensis*, or fungal organisms.

### Recruitment and consent

Potentially eligible patients were identified through microbiology laboratory reports of positive blood cultures. The site research coordinator screened the medical records of these patients to confirm that they met all inclusion criteria, and no exclusion criteria, and then provided patients with study information materials. Consenting patients could be enrolled any time up to the seventh day of adequate antibiotic treatment [10].

### Primary feasibility outcomes and secondary clinical outcomes

As per the original BALANCE-ICU pilot RCT, the co-primary feasibility outcomes were (1) recruitment rates and (2) adherence to treatment protocol. Protocol adherence was defined as receipt of 7 ± 2 days of antibiotics or 14 ± 2 days of antibiotics for patients randomized to shorter versus longer duration treatment, respectively. We did not target a specific protocol adherence rate to consider the trial feasible, but sought to determine whether the protocol adherence rate would exceed the rate seen in the BALANCE ICU pilot RCT (77%) [9]. As with the BALANCE ICU pilot RCT, we expected that there would be some patients for whom clinicians would continue antibiotic treatment beyond the assigned duration because of concerns of new infection, persistent infection, or previously unrecognized deep-seated infection. These were counted as protocol deviations. The target recruitment rate was an average of one patient per site per month to consider including ward enrolments in the BALANCE main trial. The panel of secondary clinical outcomes (e.g., length of stay, mortality, antibiotic-free days, *Clostridiodes difficile*, and antibiotic resistant organisms) were identical to those collected in the original BALANCE pilot RCT [9, 10]. Included among these secondary outcomes was the planned primary outcome from the main BALANCE RCT, 90-day mortality. Antibiotic-free days were calculated as the number of days alive and not on any antibiotics in the time period from collection of the index blood culture to 28 days after this date; patients that died prior to day 28 were assigned 0 antibiotic-free days. Treatment adherence and clinical outcomes were recorded by the site research coordinator, via chart review and discussion with the clinical team if needed.

### Data collection and follow-up

Patients were followed throughout the hospital stay to a 90-day maximum, with capture of baseline characteristics and outcome information on the same electronic case report form used for the BALANCE main trial. Ninety-day mortality was collected via follow-up phone call 90 days from the index bacteremia.

### Statistical analysis

There were no interim analyses or stopping rules within this pilot RCT. As with our initial BALANCE pilot RCT, we planned a priori to maintain blinding of treatment assignment in the BALANCE-Ward pilot RCT [11]. A feasibility pilot RCT is not powered to identify clinically important differences in safety or efficacy endpoints, but rather this is the goal of the BALANCE main RCT. We analyzed the BALANCE-Ward pilot RCT results as a single cohort, describing overall rates of recruitment per site per month and overall protocol adherence as the co-primary feasibility outcomes of interest.

Next, we compared these feasibility outcomes to those achieved during our initial BALANCE-ICU pilot RCT [9]. Poisson regression was used to compare recruitment rates per month in the ICU versus non-ICU pilots; chi-square test was used to compare protocol adherence. To further evaluate the difference between the two pilot RCTs we compared baseline patient characteristics, pathogens, foci of infection, and clinical outcomes among ward and ICU patients; the chi-square test or Fischer’s exact test were used to compare categorical variables, while a *t*-test or the Wilcoxon rank sum test were used to compare continuous variables. The Wilson Score method was used to determine 95% confidence intervals. *P* values were not adjusted for multiple comparisons.

If the BALANCE-Ward pilot demonstrated feasibility, we planned to consider merging the ward-based protocol with the ICU-based protocol of the BALANCE main trial. Therefore, we estimated the percentage of recruited patients that would be enrolled from ICU versus non-ICU wards as a function of the percentage of sites expanding to hospital-wide enrolments. Next, we estimated the impact on overall trial sample size and non-inferiority margins as a function of the proportion of anticipated ICU versus ward enrolments at the time of trial completion. For these calculations we estimated the 90-day mortality for ward patients using outcome data from this ward pilot RCT, and we estimated the mortality for ICU patients from up-to-date data from the ongoing BALANCE main RCT. At the time the ward pilot was completed, 600 patients had been enrolled and reached the 90-day endpoint in the BALANCE main trial.

### Sample size calculation

We sought to enroll a minimum of 115 patients (to equal the sample size of our BALANCE-ICU pilot) [9], but to improve generalizability of the BALANCE-Ward pilot trial we planned to continue enrolment until successful enrolment of at least one patient at all five additional non-central study sites. Recruitment extended from 17 October 2016 to 12 December 2018.

## Results

### Screened, eligible, and randomized patients

**Fig. 1:**
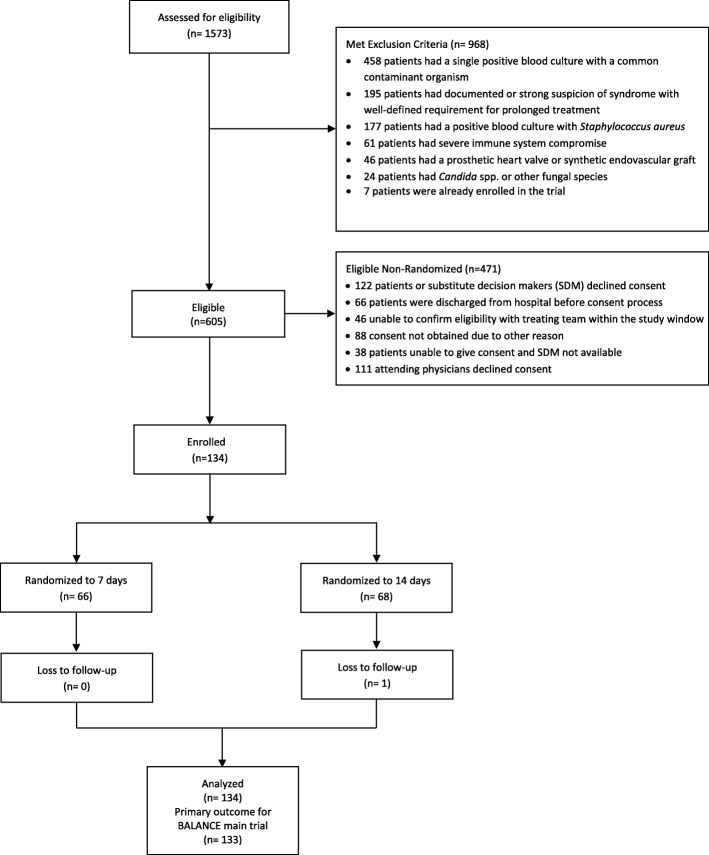
CONSORT flow diagram describing eligibility screening and randomization assignments

A total of 1573 non-ICU patients diagnosed with bacteremia on hospital wards were screened for study eligibility, of whom 605 (38%) were deemed eligible for enrolment (Fig. 1). The most common reasons for non-eligibility among the 968 excluded patients were single positive cultures with contaminant organisms (458), syndromes with well-defined requirement for prolonged treatment (195), and *S. aureus* bacteremia (177). Of eligible patients, 134/605 (22%) were enrolled and randomized (Fig. 1); this percentage ranged from 3 to 57% across participating sites (Table 1).

**Table 1:** BALANCE-Ward pilot randomized clinical trial feasibility outcomes, overall and by site

Site	Hospital type (bed size)	Months participating	Number screened	Number eligible	Number (%) enrolled	Recruitment rate (/month)	Protocol adherence (%)
A	Academic (1325)	26.9	1114	425	110 (26%)	4.1	103 (94%)
B	Community (426)	3.6	28	28	16 (57%)	4.4	15 (94%)
C	Community (410)	3.7	191	86	4 (5%)	1.1	4 (100%)
D	Academic (607)	6.7	167	39	1 (3%)	0.1	1 (100%)
E	Academic (977)	1.0	60	20	1 (5%)	1.0	1 (100%)
F	Academic (440)	5.1	13	7	2 (29%)	0.4	1 (50%)
Total	4185	47.0	1573	605	134 (22%)	2.9	125 (93%)

### Recruitment rate

A total of 134 patients were recruited over 47 site-months (mean 2.9 patients/site-month; Table 1). The recruitment rate varied across the six participating sites: hospital A (4.1 patients per month, over 26.9 months), hospital B (4.4 patients/month, over 3.6 months), hospital C (1.1 patient/month, over 3.7 months), hospital D (0.1 patients per month, over 6.7 months), hospital E (1.0 patients/month, over 1 month), and hospital F (0.4 patients/month, over 5.1 months) (Table 1). The overall recruitment rate significantly exceeded the recruitment rate in the BALANCE-ICU pilot RCT (2.9 patients/site-month vs 1.1 patients/site-month, *p* < 0.0001).

### Protocol adherence

The overall adherence to treatment duration protocol was 125/134 (93%), with minimal variation across study sites: SHSC 103/110, SJHC 15/16, TOH 1/1, KGH 1/2, NYGH 4/4, HGH 1/1 (Table 1). Overall protocol adherence significantly exceeded the adherence achieved in the BALANCE-ICU pilot RCT (125/134, 93% vs 89/115, 77%, *p* = 0.0003).

### Patient, infection, and pathogen characteristics

Patients enrolled in the BALANCE-Ward pilot RCT were older than those enrolled in the ICU pilot RCT (median (IQR) 72(62–82) vs 67(57–78) years, *p* = 0.010), but had a lower Sequential Organ Failure Assessment (SOFA) score (2(0–3) vs 6 (4–9), *p* < 0.0001) on the day blood cultures were collected (Table 2). A greater proportion of the bacteremias in non-ICU ward patients were community-acquired (84 vs 60%, *p* < 0.0001), and a greater proportion were due to genito-urinary sources of infection (49 vs 23%, *p* < 0.0001) and/or *E. coli* as a causative pathogen (49 vs 24%, *p* < 0.0001) (Table 2). However, a broad variety of pathogens was still implicated in the non-ICU infections (30 pathogens among the 134 patients), and the top ten pathogen list was similar to the top pathogens seen in the BALANCE-ICU pilot RCT (Table 2).

**Table 2:** Patient, pathogen, and infection characteristics in the BALANCE-Ward pilot RCT compared to the BALANCE-ICU pilot RCT

Characteristic	BALANCE-Ward pilot *n* = 134	BALANCE-ICU pilot *n* = 115	*P* value
Patient characteristic			
Male sex	65 (49)	63 (55)	0.32
Age in years	72 (62–82)	67 (57–78)	0.01
SOFA score on day 0	2 (0–3)	6 (4–9)	< 0.001
Comorbidity^a^			
Coronary artery disease	19 (14)	23 (20)	0.22
Congestive heart failure	11 (8)	16 (14)	0.15
Arrhythmia	18 (14)	15 (13)	0.93
Peripheral vascular disease	6 (5)	14 (12)	0.03
Diabetes mellitus	31 (23)	40 (35)	0.04
Renal insufficiency	16 (12)	13 (11)	0.88
Dialysis dependency	3 (2)	4 (4)	0.71
Chronic obstructive pulmonary disease	7 (5)	16 (14)	0.02
Liver disease	1 (1)	8 (7)	0.01
Obesity	6 (5)	16 (14)	0.01
Solid malignancy	40 (30)	18 (16)	0.01
Leukemia/lymphoma	6 (5)	1 (1)	0.13
Corticosteroid use/immunosuppression	22 (17)	10 (9)	0.07
Infection characteristics			
Acquisition of bacteremia			< 0.001
Community-acquired	113 (84)	69 (60)	
Hospital-acquired	21 (16)	46 (40)	
Source of bacteremia			< 0.001
Lung	9 (7)	31 (27)	
Intra-abdominal/hepato-biliary	23 (17)	29 (25)	
Urinary tract	65 (49)	26 (23)	
Vascular-catheter related	7 (5)	9 (8)	
Skin and/or soft tissue	4 (3)	4 (3)	
Other	6 (4)	4 (3)	
Undefined/unknown	20 (15)	12 (10)	
Most commonly isolated pathogens in blood cultures^b^			
*Escherichia coli*	65 (49)	28 (24)	< 0.001
*Klebsiella* spp*.*	19 (14)	18 (16)	0.74
*Enterococcus* spp*.*	6 (4)	17 (15)	0.01
*Streptococcus pneumonia*	8 (6)	13 (11)	0.13
Coagulase negative staphylococci	1 (1)	10 (9)	0.002
*Enterobacter* spp*.*	8 (6)	6 (5)	0.80
*Pseudomonas* spp*.*	4 (3)	4 (3)	1.00
*Serratia* spp*.*	3 (2)	4 (3)	0.71
*Citrobacter* spp*.*	2 (1)	3 (3)	0.66
*Streptococcus anginosus* group	2 (1)	3 (3)	0.66

All data are presented as *n* (%) or medians (interquartile ranges) unless otherwise specified

^a^ One patient in the Ward-pilot group and one patient in the ICU-pilot group have unknown comorbidities

^b^ A total of 32 different bacterial species were isolated among the index blood cultures of the 115 ICU patients; a total of 30 different species were isolated among the 134 ward patients

*SOFA* Sequential Organ Failure Assessment

### Clinical outcomes

As per a priori plans, we did not examine clinical outcomes separated by treatment duration arm in this pilot RCT. The BALANCE-Ward pilot RCT patients had an overall 90-day mortality rate of 17/133 (12.8%, 95% CI 8.1–19.5%), which was similar to the 90-day mortality rate in the ICU pilot RCT (17/115, 14.8%, 95% CI 9.4–22.4%) (*p* = 0.65; Table 3) and mortality estimates from the main BALANCE RCT as of 600 patients enrolled (104/600, 17.3%, 95% CI 14.5–20.6%). The patients in the BALANCE-Ward pilot had a shorter median (IQR) length of hospital stay (6 (4–12) vs 20(12–43) days, *p* < 0.001) and more antibiotic-free days by day 28 (14(14–21) vs 14(8–17), *p* < 0.0001) (Table 3). Only one patient was lost to follow-up at 90 days, but there are ongoing efforts to ascertain final vital status for this patient.

**Table 3:** Clinical outcomes in the BALANCE-Ward pilot RCT compared to the BALANCE-ICU pilot RCT and updated data from the BALANCE main RCT

Outcome	BALANCE Ward Pilot *n* = 134	BALANCE ICU pilot *n* = 115	BALANCE Main RCT^a^ *n* = 600	*P* value Ward pilot vs ICU pilot	*P* value Ward pilot vs main RCT
Mortality					
In hospital	3/134 (2)	15/115 (13)	95/597 (16)	0.001	< 0.001
At 90 days^b^	17/133 (13)	17/115 (15)	104/600 (17)	0.65	0.20
Length of stay in hospital (in days)	6 (4–12)	20 (12–43)	20 (11–43)	< 0.001	< 0.001
Relapse of bacteremia	1 (1)	4 (3)	12 (2)	0.18	0.48
Antibiotic-free days (by day 28)	14 (14–21)	14 (8–17)	14 (6–18)	< 0.001	< 0.001
Antimicrobial-related adverse outcomes					
Allergy	0 (0)	0 (0)	9/598 (2)	1.00	0.38
Anaphylaxis	0 (0)	0 (0)	0/598 (0)	1.00	1.00
Acute kidney injury	0 (0)	0 (0)	1/598 (0.2)	1.00	1.00
Acute hepatitis	0 (0)	1 (1)	2/598 (0.3)	0.46	1.00
*Clostridiodes difficile* infection	0 (0)	4 (3)	12/598 (2)	0.04	0.14
Secondary infection with resistant microorganisms	11 (8)	10 (9)	72 (12)	0.89	0.18

All data are presented as medians and interquartile ranges unless otherwise specified

^a^ Based on up-to-date data from the first 600 ICU patients enrolled in the BALANCE main RCT

^b^ One patient loss to follow-up for 90-day outcome (but ongoing efforts underway to ascertain vital status)

### Modeling the final proportion of patients that would be enrolled in ICU versus non-ICU settings

**Fig. 2:**
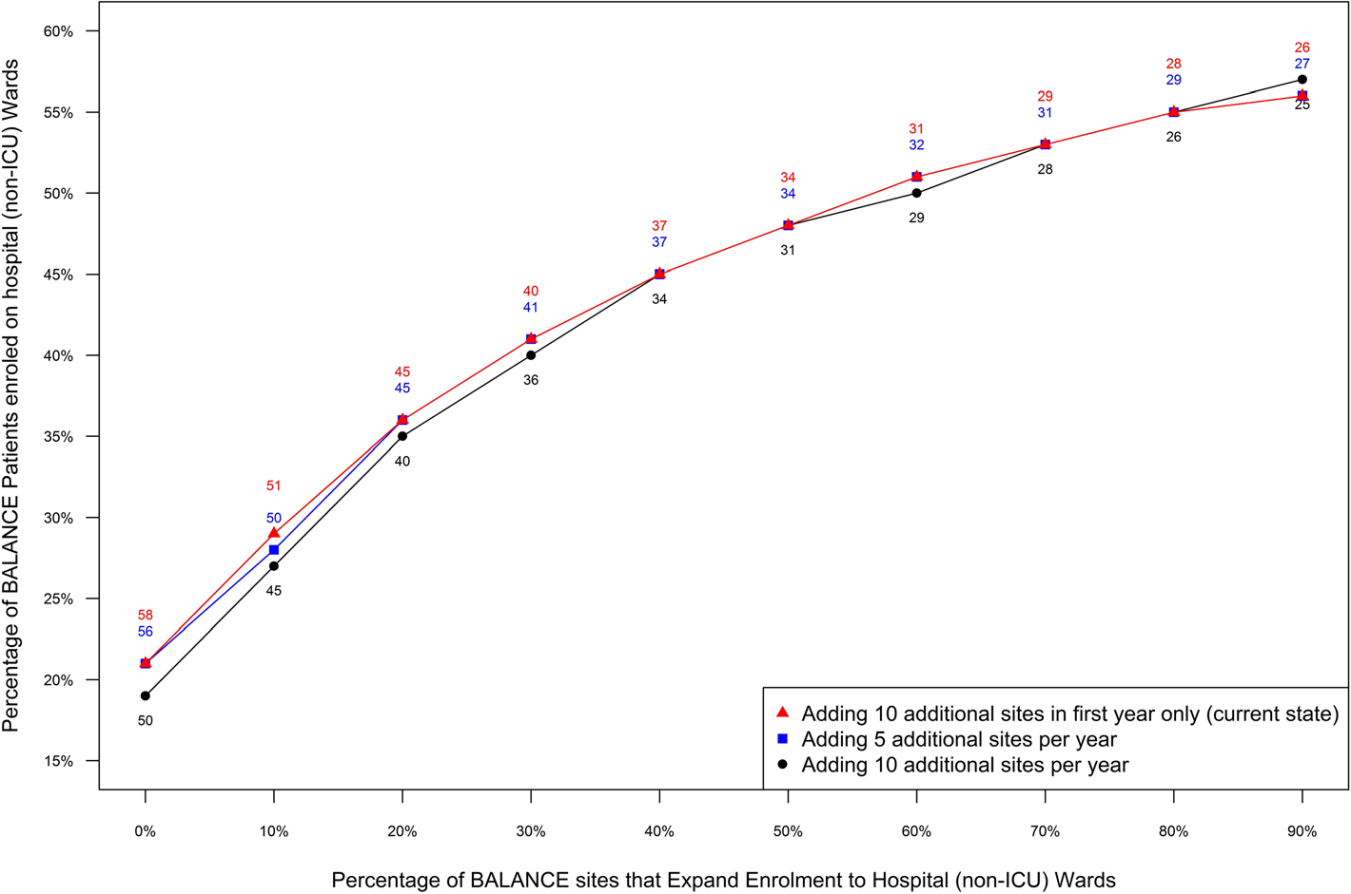
Estimating the final proportion of patients enrolled on non-ICU wards, as a function of the percentage of participating sites which expand to include non-ICU ward enrolments. This analysis assumes average rates of enrolment in ICU based on current BALANCE trial data and in non-ICU wards based on the BALANCE-Ward pilot. The colored lines depict projections accounting for current number of registered sites (*red*), as well as under assumptions of adding additional sites (five per year, *blue*; ten per year, *black*) over the duration of the trial. The projected number of months remaining until trial completion are listed above each data point

Assuming average enrolment rates in the ICU based on up-to-date data from the BALANCE main trial, as well as ward enrolment rates from this BALANCE-Ward pilot RCT, we are able to estimate how the final proportion of ICU versus non-ICU patients will vary according to the proportion of sites that choose to expand enrolment onto non-ICU wards (Fig. 2). Even under scenarios in which three-quarters of sites expand to non-ICU wards, the final study population will still be comprised of nearly half ICU patients (Fig. 2).

### Modeling sample size and non-inferiority margin implications of merging non-ICU ward patients into the BALANCE main RCT

**Fig. 3:**
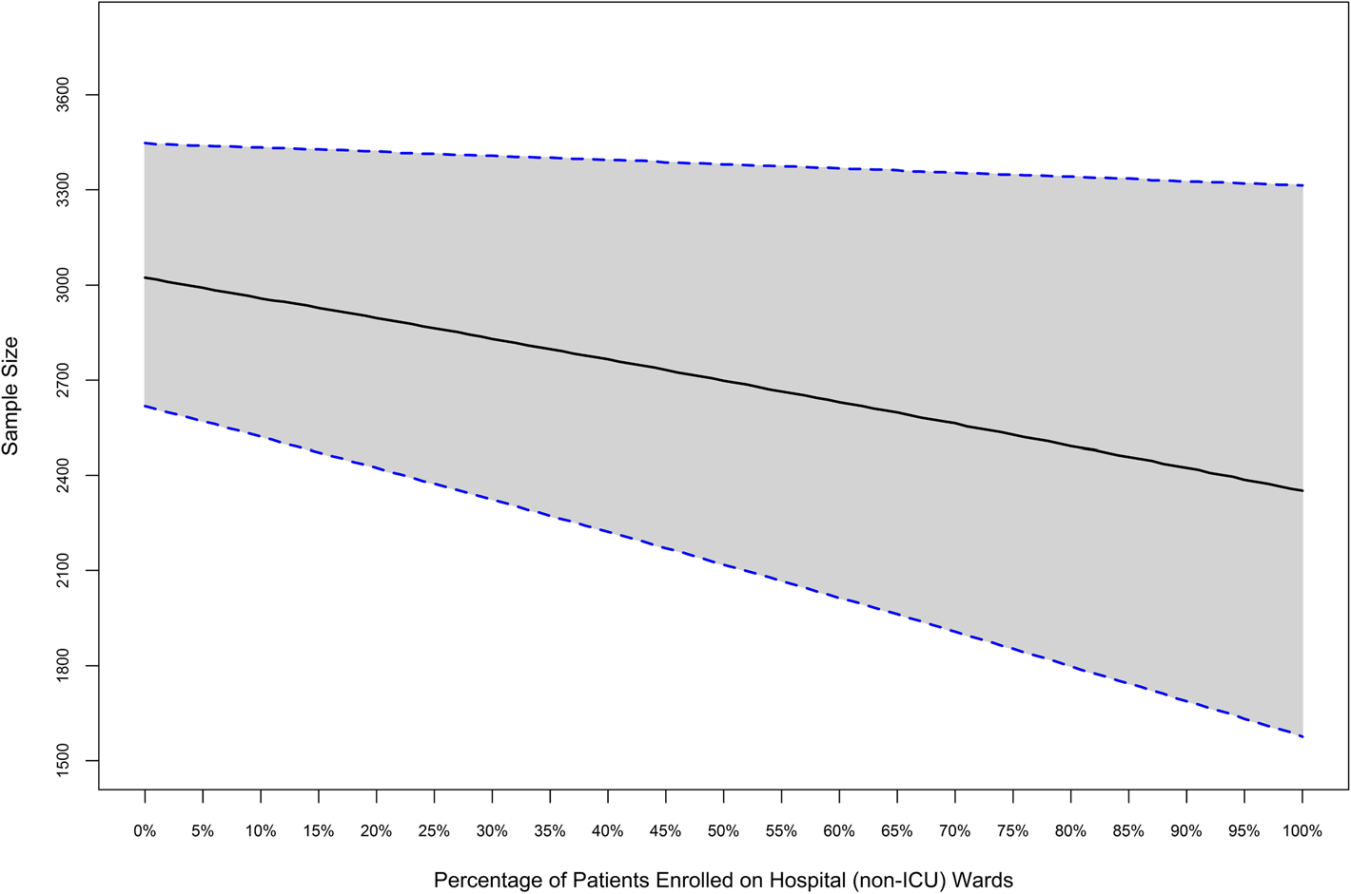
Sample size implications of expanding the BALANCE main RCT to include non-ICU ward patients, as a function of the final percentage of patients enrolled from non-ICU wards and fixing the non-inferiority margin at 4%. The point estimates (*solid black line*) assume a mortality rate of 17.3% among ICU patients and 12.8% in non-ICU ward patients, with 95% certainty estimates around those estimates (*gray shaded area*)

**Fig. 4:**
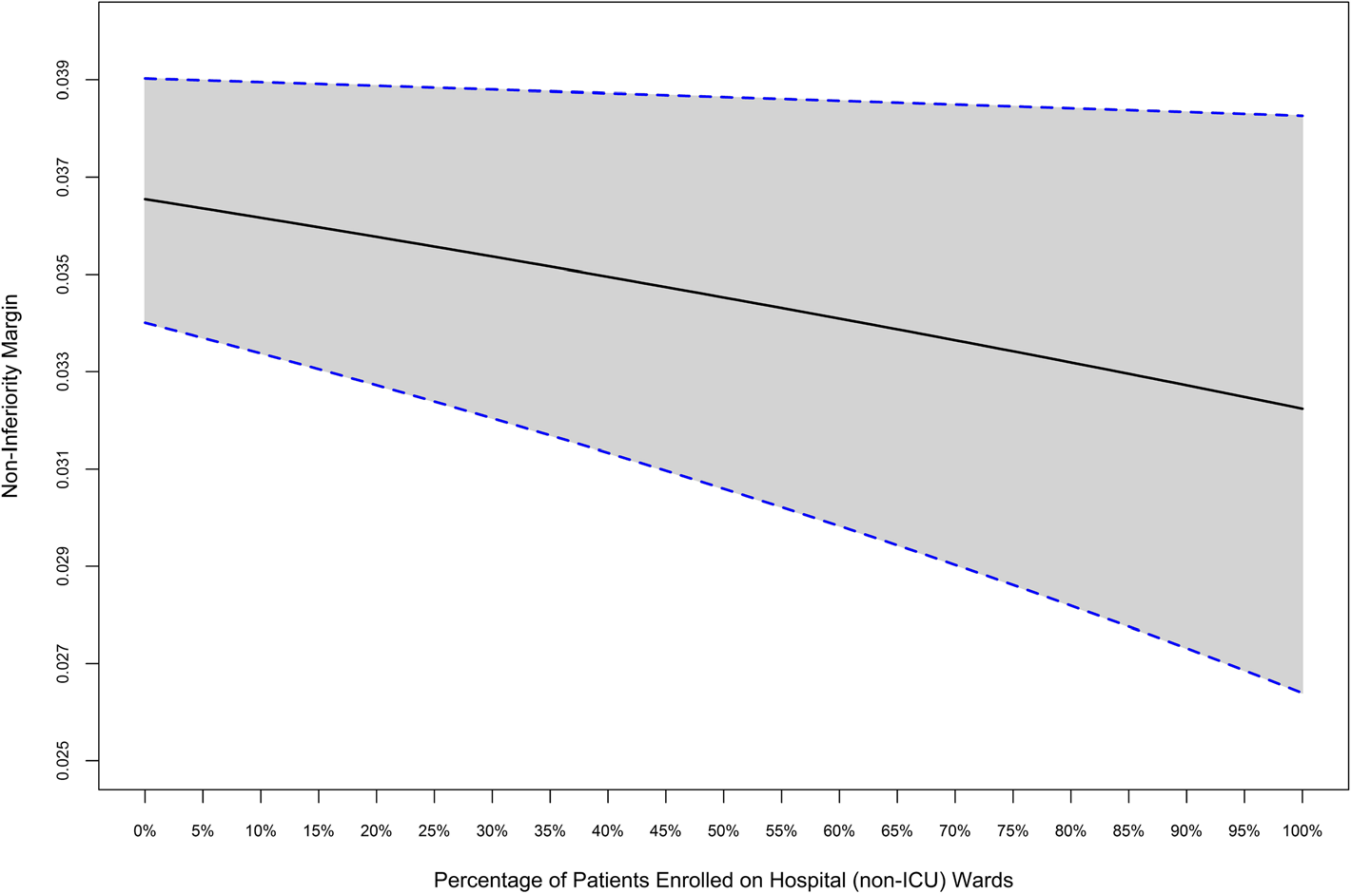
Non-inferiority margin implications of expanding the BALANCE main RCT to include non-ICU ward patients, as a function of the final percentage of patients enrolled from non-ICU wards and fixing the sample size at 3626. The point estimates (*solid black line*) assume a mortality rate of 17.3% among ICU patients and 12.8% in non-ICU ward patients, with 95% certainty estimates around those estimates (*gray shaded area*)

Assuming a 90-day mortality rate of 12.8% among BALANCE ward patients and 17.3% among BALANCE-ICU patients (based on most up-to-date data from the main BALANCE trial), merging ward patients into the main trial would result in an overall mortality rate of 15% if there were equal numbers of ward and ICU patients. Figures 3 and 4 depict the sample size and non-inferiority margin implications of merging ward patients into the BALANCE RCT as a function of the final percentage of ward patients enrolled.

## Discussion

In the prior BALANCE-ICU pilot RCT we demonstrated that it was feasible to enroll ICU patients into a trial of 7 versus 14 days of treatment for bloodstream infection [9], thereby providing the vanguard patients for the multinational, multicentre BALANCE main RCT. In this subsequent BALANCE-Ward pilot RCT, we have confirmed that it is feasible to enroll patients cared for on general hospital wards and have clarified the viability and implications of expanding the BALANCE main RCT to include hospital-wide patients with bacteremia.

The BALANCE-Ward pilot RCT documented feasibility with respect to both co-primary outcomes of recruitment rate and protocol adherence. We achieved mean recruitment rates of 2.9 patients per site-month; the median recruitment rate per site per month was lower (1.0) but still met our feasibility target. Similarly, we achieved protocol adherence rates of 93%, which exceeded the 77% adherence rates in the ICU population. On the basis of these co-primary outcomes it appears feasible that the BALANCE RCT could be extended from ICUs to include non-ICU patients. The increased recruitment rate on the wards can be attributed to the larger number of bacteremic patients than those who are in the ICU. The superior protocol adherence rates on the general wards may be due to the lower severity of illness and lower risk of secondary nosocomial infections among these patients with shorter lengths of hospital stay and fewer indwelling devices such as endotracheal tubes and central venous catheters.

As expected, there were some measurable differences in critically ill patients with bacteremia enrolled in the initial BALANCE pilot compared to the patients on the wards who were enrolled in this pilot. The latter were older, had lower severity of illness at baseline, and more commonly had community-acquired bacteremia, genito-urinary sources of infection, and *E. coli* as a causative pathogen. On the one hand, merging non-ICU patients with ICU patients into a single trial could be viewed as mixing two heterogeneous populations together. On the other hand, combining these patients together could be considered as reflecting a broader population of patients with bloodstream infection, yielding more generalizable trial results. The ICU and non-ICU pilot trial patients were both infected with a diverse range of Gram negative and Gram positive bacterial pathogens, and each included patients with a diverse range of host comorbidities. Typically a trial based on a specific diagnosis (e.g., pulmonary embolism, myocardial infarction) would be conducted across the full spectrum of severity, including those patients admitted to ICU and non-ICU wards. Conceptually, enrolling both non-ICU and ICU patients captures the full spectrum of bacteremic illness, and the patients are only dichotomized by the location of care within the hospital.

The 90-day mortality rate in this pilot RCT (12.8%) was similar to the mortality rate seen in a recently published RCT of 604 patients allocated to 7 versus 14 days of antibiotics for patients with Gram negative bacteremia conducted on non-ICU wards in three centers in Israel and Italy [12]. As expected, the 90-day mortality rate was lower than that seen in our prior BALANCE-ICU pilot RCT (15%) [9]. The mortality difference between non-ICU and ICU patients is even wider than the ICU pilot data suggest, because a more updated mortality estimate from the BALANCE main trial suggests that the mortality has risen to 17.3%. At a fixed non-inferiority margin of 4%, adding non-ICU ward patients in the study would decrease our total sample size requirement (Fig. 3); maintaining our sample size target would enable us to reduce the achievable absolute non-inferiority margin (Fig. 4). It is important to note that our 4% non-inferiority margin is already much smaller than the non-inferiority margins used in recent trials of antibiotic treatment duration in patients with serious bacterial infections [12–15], and is also much lower than the US Federal Drug Administration recommendation of non-inferiority margins for ventilator-associated pneumonia [16]. Therefore, we have opted to maintain our current overall sample size target (*n* = 3626) for the BALANCE main trial.

Our BALANCE-Ward pilot RCT enrolled patients in six sites, and so we cannot be certain that the recruitment and adherence results would be generalizable to all of the sites involved in the BALANCE main RCT. However, the generalizability is bolstered by inclusion of a mix of both community and academic hospitals, as well as sites with long-standing versus recent involvement in the CCCTG. Another limitation is that we cannot predict whether expansion to include non-ICU enrolment will lead to a compensatory decrease in ICU recruitments by diluting study teams’ efforts across broader clinical units. In our six pilot RCT sites, though, we did not see reductions in ICU recruitments. As BALANCE is expanded hospital-wide, we will assess the interplay of ICU and non-ICU recruitment rates over time. The low rate of enrolment of eligible ward patients, and wide variation across sites, suggests that further efforts may be necessary to foster enrolments, including educating ward clinicians about the pre-RCT work which has documented practice heterogeneity and collective clinical equipoise. The BALANCE-Ward pilot RCT experience suggests that sites with infectious diseases engagement on the study team achieve much higher recruitment rates and percent enrolment of eligible patients, and so this will be crucial for future sites considering hospital-wide recruitment. We will also need to track eligible non-enrolled patients, along with recruitment rates and protocol adherence, as a site-specific metric throughout the conduct of the trial.

The BALANCE steering committee and CCCTG have guided us in conducting step-wise pilots of the BALANCE RCT protocol in the initial ICU population, and now in this non-ICU population, once again confirming the feasibility of the BALANCE trial design on general hospital wards. We have carefully reviewed the one-group findings (maintaining allocation concealment) with the CCCTG and the BALANCE international steering committee, both of which have strongly endorsed the option for participating BALANCE sites to extend enrolments hospital-wide. Given the success of this non-ICU pilot, no other protocol changes are required to facilitate inclusion of non-ICU patients in the BALANCE main trial. A detailed statistical analysis plan involving the entire cohort will be published before the trial is completed; randomization will be stratified by ICU and non-ICU ward location, and a subgroup analysis will be conducted. The subgroup analyses, by definition, will not be powered to achieve the same non-inferiority margin as the overall BALANCE trial population. However, the achievable non-inferiority margins within the ICU and non-ICU subgroups will still be less than the non-inferiority margins used in recent landmark antimicrobial minimization studies involving patients with serious bacterial infections [4, 13–15]. We anticipate that the final BALANCE trial results will be more generalizable to the full population of patients admitted to hospital with bloodstream infections, and yet will include a majority of critically ill patients, ensuring that the data are relevant to our sickest of patients. In doing so, we hope that BALANCE will provide an evidence foundation for the treatment of a broad range of patients with non-*S. aureus* bacteremia, and allow us to maximize the benefits while minimizing the harms of antimicrobial treatments for bloodstream infections.

## Data Availability

The datasets used and/or analyzed during the current study are available from the corresponding author on reasonable request.

## Abbreviations

ANZICS CTG: Australian & New Zealand Intensive Care Society Clinical Trials Group
BALANCE: Bacteremia Antibiotic Length Actually Needed for Clinical Effectiveness
CCCTG: Canadian Critical Care Trials Group
ICU: Intensive care unit
IQR: Interquartile range
RCT: Randomized clinical trial
SOFA: Sequential Organ Failure Assessment
